# Impact of HIV infection on the presentation, outcome and host response in patients admitted to the intensive care unit with sepsis; a case control study

**DOI:** 10.1186/s13054-016-1469-0

**Published:** 2016-10-10

**Authors:** Maryse A. Wiewel, Michaëla A. Huson, Lonneke A. van Vught, Arie J. Hoogendijk, Peter M. C. Klein Klouwenberg, Janneke Horn, René Lutter, Olaf L. Cremer, Marcus J. Schultz, Marc J. Bonten, Tom van der Poll, on behalf of the MARS Consortium, Friso M. de Beer, Lieuwe D. J. Bos, Jos F. Frencken, Gerie J. Glas, Roosmarijn T. M. van Hooijdonk, David S. Y. Ong, Laura R. A. Schouten, Brendon P. Scicluna, Marleen Straat, Esther Witteveen, Luuk Wieske

**Affiliations:** 1 Center for Experimental and Molecular Medicine, Academic Medical Center, University of Amsterdam, Amsterdam, The Netherlands; 2 The Center for Infection and Immunity Amsterdam, Academic Medical Center, University of Amsterdam, Amsterdam, The Netherlands; 3 Department of Intensive Care, Academic Medical Center, University of Amsterdam, Amsterdam, The Netherlands; 4 Department of Respiratory Medicine and Experimental Immunology, Academic Medical Center, University of Amsterdam, Amsterdam, The Netherlands; 5 Division of Infectious Diseases, Academic Medical Center, University of Amsterdam, Amsterdam, The Netherlands; 6 Department of Intensive Care Medicine, University Medical Center Utrecht, Utrecht, The Netherlands; 7 Department of Medical Microbiology, University Medical Center Utrecht, Utrecht, The Netherlands; 8 Julius Center for Health Sciences and Primary Care, University Medical Center Utrecht, Utrecht, The Netherlands

**Keywords:** HIV, Sepsis, Pneumonia, Intensive care units

## Abstract

**Background:**

Sepsis is a prominent reason for intensive care unit (ICU) admission in patients with HIV. We aimed to investigate the impact of HIV infection on presentation, outcome and host response in sepsis.

**Methods:**

We performed a prospective observational study in the ICUs of two tertiary hospitals. For the current analyses, we selected all patients diagnosed with sepsis within 24 hours after admission. Host response biomarkers were analyzed in a more homogeneous subgroup of admissions involving HIV-positive patients with pneumosepsis, matched to admissions of HIV-negative patients for age, gender and race. Matching was done by nearest neighbor matching with R package “MatchIt”.

**Results:**

We analyzed 2251 sepsis admissions including 41 (1.8 %) with HIV infection (32 unique patients). HIV-positive patients were younger and admission of HIV-positive patients more frequently involved pneumonia (73.2 % versus 48.8 % of admissions of HIV-negative patients, *P* = 0.004). Disease severity and mortality up to one year after admission did not differ according to HIV status. Furthermore, sequential plasma levels of host response biomarkers, providing insight into activation of the cytokine network, the vascular endothelium and the coagulation system, were largely similar in matched admissions of HIV-positive and HIV-negative patients with pneumosepsis.

**Conclusions:**

Sepsis is more often caused by pneumonia in HIV-positive patients. HIV infection has little impact on the disease severity, mortality and host response during sepsis.

**Electronic supplementary material:**

The online version of this article (doi:10.1186/s13054-016-1469-0) contains supplementary material, which is available to authorized users.

## Background

The spectrum of disease in human immunodeficiency virus (HIV)-infected patients has changed dramatically since the introduction of combination antiretroviral therapy (cART) [1]. The incidence of opportunistic infections has decreased and long-term survival has improved to an extent that HIV infection has become a chronic disease [1]. However, invasive bacterial infections and sepsis remain an important cause of morbidity and mortality in patients with HIV [2, 3], and previous studies have demonstrated the importance of sepsis as a reason for intensive care unit (ICU) admission [3–8]. Advanced HIV infection has been associated with higher mortality in patients with sepsis compared to mortality in HIV-negative patients with sepsis [9–11].

Sepsis is characterized by an imbalanced host response, characterized amongst other factors by release of proinflammatory and anti-inflammatory cytokines, activation of the vascular endothelium and stimulation of the coagulation system with concurrent impairment of anticoagulant mechanisms [12]. HIV infection is associated with activation and deregulation of several cellular and mediator pathways also implicated in the pathogenesis of sepsis, which has led to the hypothesis that HIV infection may further disturb the host response in sepsis [13]. However, few studies have investigated the immune response to sepsis in patients with HIV co-infection.

We aimed to compare the presentation and outcome of sepsis in the presence or absence of HIV co-infection in an area with widely available cART. In addition, in a more homogeneous subgroup of patients with pneumosepsis, we sought to obtain insight into the influence of HIV co-infection on the host response.

## Methods

### Study design, patients and definitions

This study was conducted as part of the Molecular Diagnosis and Risk Stratification of Sepsis (MARS) project, a prospective observational study in the ICUs of two tertiary teaching hospitals (Academic Medical Center in Amsterdam and University Medical Center Utrecht, The Netherlands) [14]. Trained ICU researchers prospectively collected demographic, clinical, microbiological and interventional data [14]. In the MARS study, assignment of pathogens to pneumonia cases was based on post hoc physician assessment, using all available information, including pathogens cultured from blood, lower respiratory tract samples and respiratory secretions and serology and PCR results, combined with the clinical decision to treat the patient for the particular causative pathogen, in multidisciplinary meetings.

Information on CD4 counts, viral loads and cART was collected from patient files. CD4 counts and viral loads measured between 120 days prior to and 30 days after admission were considered representative. If multiple samples were available, the first sample was included in our analyses. Organ failure was defined by a score of 3 or greater on the Sequential Organ Failure Assessment (SOFA) score, or a score of 1 or more for cardiovascular failure [15]. Shock was defined by the use of vasopressors (noradrenaline) for hypotension in a dose of 0.1 mcg/kg/min during at least 50 % of the ICU day. The plausibility of infection was assessed post hoc and classified on a 4-point scale (none, possible, probable or definite) according to Center for Disease Control and Prevention [16] and International Sepsis Forum consensus definitions [14, 17].

For the current analysis we selected all patients admitted to the ICU between January 2011 and July 2013 with sepsis diagnosed within 24 hours after ICU admission, defined as the presence of infection combined with at least one additional parameter as described in the 2001 International Sepsis Definitions Conference [18]. Patients with a post hoc infection likelihood of “none” were excluded, as were patients transferred from another ICU, except for those referred to one of the study centers on the day of admission. The Municipal Personal Records Database was consulted to determine survival up to one year after ICU admission. In The Netherlands, all deaths are immediately reported to this database, so this provides a reliable and up-to-date means to assess mortality. If a patient had multiple admissions, only the first admission was used to assess mortality.

### Biomarker measurements

Daily (at admission and at 6 a.m. thereafter) left-over plasma (obtained from blood drawn for routine patient monitoring) was stored within 4 hours at -80 °C. All measurements were done in EDTA anticoagulated plasma obtained on admission (day 0) and days 2 and 4. Tumor necrosis factor alpha (TNF-α), interleukin-1beta (IL-1β), interferon-gamma (IFN-γ), IL-6, IL-8, IL-10, IL-13, soluble intercellular adhesion molecule-1 (ICAM-1) and soluble E-selectin were measured using FlexSet cytometric bead arrays (BD Bioscience, San Jose, CA, USA) using FACS Calibur (Becton Dickenson, Franklin Lakes, NJ, USA). Angiopoietin-1, angiopoietin-2, protein C, antithrombin (R&D systems, Abingdon, UK), and D-dimer (Procartaplex, eBioscience, San Diego, CA, USA) were measured by Luminex multiplex assay using BioPlex 200 (BioRad, Hercules, CA, USA). Normal biomarker values were acquired from EDTA plasma from 27 age-matched and gender-matched healthy volunteers, from whom written informed consent was obtained.

### Statistical analysis

Data-analyses were performed in R (v3.1.1) [19]. Baseline characteristics of study groups were compared using the chi-square test for categorical variables and the *t* test or Wilcoxon rank sum test for continuous variables. In order to adjust for differences in clinical characteristics between groups, each HIV patient with pneumonia was matched by age, gender and race (white) to three HIV-negative controls, using nearest neighbor matching with R package “MatchIt”. Biomarkers were transformed to a log scale and mixed models were used to analyze repeated measurements. *P* values below 0.05 were considered statistically significant.

## Results

### Study population

A total of 6994 admissions, involving 5920 unique patients, were included during the study period, including 58 admissions of HIV-positive patients (0.8 %). We excluded 325 admissions because the patients had been transferred from other ICUs. Of the remaining 6669 admissions, 2251 (1889 patients) had a sepsis diagnosis in the first 24 hours of ICU admission, including 41 admissions (1.8 %) of 32 unique patients (1.7 %) with HIV infection (Table 1). The other 17 HIV-positive admissions (16 unique patients) during this study period were for non-infectious reasons, predominantly postoperative surveillance (n = 4), respiratory insufficiency (n = 3) and pulmonary embolism (n = 2).

**Table 1:** Baseline characteristics and outcome of all sepsis admissions stratified according to HIV infection status

	HIV-positive (n = 41)	HIV-negative (n = 2210)	*P*
Demographics^a^			
Age, years, mean (SD)	49.6 (12)	60.4 (15.5)	<0.0001
Gender, male (%)	24 (75)	1154 (6)	0.14
Race, white (%)	18 (56.2)	1652 (89)	0.005
Admissions			
Readmission (%)	9 (22.0)	353 (16.0)	0.39
New HIV diagnosis (%)	6 (14.6)	--	--
Severity of disease in first 24 hours			
SOFA score, median (IQR)^b^	7 (3-10.5)	7 (4–9)	0.38
Organ failure (%)	35 (85.4)	1802 (81.5)	1
Shock (%)	9 (22)	606 (27.4)	0.50
HIV disease severity and treatment			
CD4 count, cells/mm^3^, median (IQR)^c^	70 (23-346)	--	--
Viral load, cp/ml, median (IQR)^d^	105 (48-54916)	--	--
On cART (%)	29 (70.7)	--	--
Viral suppression (%)	18 (47.4)	--	--
Outcome			
Length of ICU stay, median days (IQR)	4 (1–11)	4 (2–8)	0.47
Organ failure during admission (%)	37 (90.2)	1915 (86.7)	0.73
Shock during admission (%)	15 (36.6)	750 (33.9)	0.74
30-day mortality (%)^a^	6 (18.8)	481 (25.9)	0.42
60-day mortality (%)^a^	11 (34.4)	568 (30.6)	0.72
90-day mortality (%)^a^	13 (40.6)	629 (33.9)	0.57
1-year mortality (%)^a^	16 (50)	794 (42.8)	0.35

^a^Demographic and mortality data are given for the first ICU admission during the study period; readmissions were not included, resulting in analysis of 32 HIV-positive patients and 1857 HIV-negative patients. From the total of 2251 admissions, 23 were lost to follow up at day 30 (1 %), 32 at day 60 (1.4 %), 37 at day 90 (1.6 %), and 59 at 1 year (2.6 %) after ICU admission. ^b^The central nervous system score was excluded from the Sequential Organ Failure Assessment (SOFA) score calculation, because of a large number of sedated patients. ^c^CD4 counts were available for 39 admissions. In 32 patients (82 %) the CD4 count was obtained within 120 days prior to admission, and 7 patients (18 %) had a CD4 count obtained on admission or within 30 days after admission. ^d^Viral loads were available for 39 admissions. Viral suppression was defined as a viral load below the detection limit, which was <40 copies/ml or <50 copies/ml, depending on the hospital laboratory. *cART* combination antiretroviral therapy, *IQR* interquartile range, *SD* standard deviation

### Presentation, cause and outcome of sepsis

**Fig. 1:**
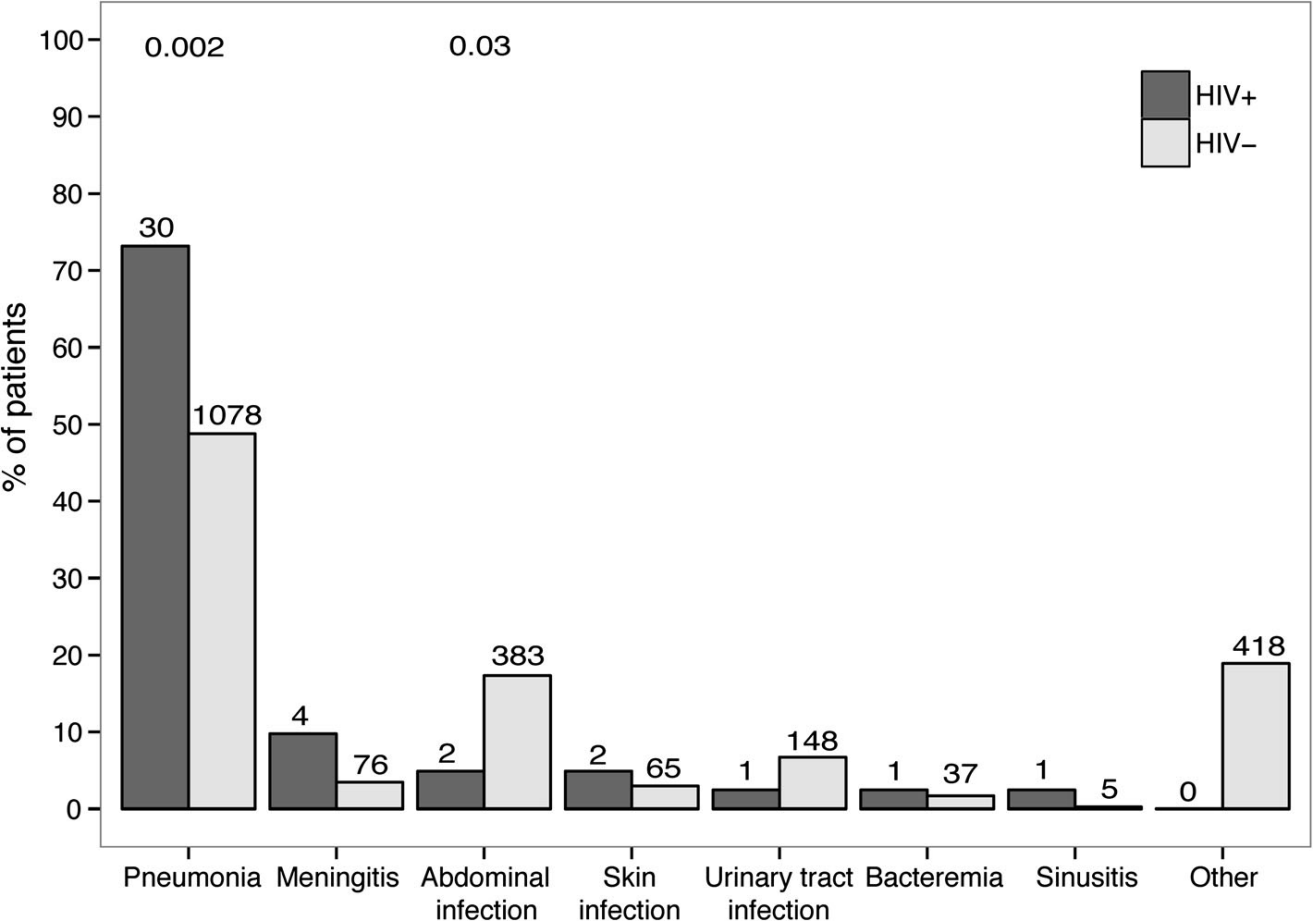
Primary sites of infection in patients with sepsis admitted to the ICU stratified by HIV status. *Numbers* in the figure represent the numbers of patients per group. *P* values are indicated for differences between HIV-positive and HIV-negative patients at each time point

Sepsis admissions with HIV co-infection involved younger patients, who were less likely to be Caucasian, compared to sepsis admissions without HIV infection (Table 1). The majority of HIV-positive patient admissions (n = 29, 70.7 %) involved patients who were on cART, but only 18 (47.4 %) had complete viral suppression (HIV load <50 copies/ml). There were 6 admissions (14.6 %) involving patients presenting with newly diagnosed HIV infection and the majority of HIV admissions presented with overt immune suppression (CD4 counts <200 cells/mm^3^ in 56.1 % and <350 cells/mm^3^ in 73.2 % of admissions). Pneumonia was the most common infection in both HIV-positive and HIV-negative admissions, but pneumonia was more frequent in HIV-positive patient admissions (n = 30 (73.2 %) versus 1048 (48.8 %) in admissions involving patients with sepsis who were HIV-negative, *P* = 0.004) (Fig. 1). The proportion of patients admitted with organ failure or shock was similar in HIV-positive and HIV-negative patients (Table 1). Likewise, the occurrence of organ failure and shock at any day during ICU stay was similar between groups. Crude mortality up to one year after ICU admission did not differ between HIV-positive and HIV-negative patients with sepsis.

### Presentation, cause and outcome of pneumonia

Considering the strong predominance of pneumonia amongst HIV-positive admissions, we focused our further analyses on 30 admissions of HIV-positive patients with pneumosepsis (Table 2). Considering the large demographic differences according to HIV status, we composed a control cohort of 90 admissions of HIV-negative patients with pneumonia, matched for age, sex and race. In the matched cohort demographic characteristics were similar between groups. The matched cohort contained more HIV-positive than HIV-negative patients who were readmitted. In order to explore reasons for the relatively high readmission rates amongst HIV-positive patients we compared HIV-positive admissions with and without readmission (Additional file 1); this analysis did not provide a clear explanation. Although the severity of disease was comparable between HIV-positive and HIV-negative admissions, as reflected by the SOFA score and the percentage of patients presenting with organ failure or shock, HIV-positive pneumosepsis admissions were significantly less likely to require mechanical ventilation in the first 24 hours. Causative pathogens in patients with pneumosepsis are outlined in Table 3. The most common pathogens in HIV-positive admissions were *Streptococcus pneumoniae*, *Staphylococcus aureus* and *Pneumocystis jirovecii*. While *S. pneumoniae* and *S. aureus* were similarly frequent in HIV-negative patients, *P. jirovecii* was more common in HIV-positive patients compared with unmatched HIV-negative patients with pneumosepsis, but not when compared to matched HIV-negative patients with pneumosepsis. In the latter group *P. jirovecii* pneumonia occurred in patients on immunosuppressive therapy. Cytomegalovirus (CMV) was a more frequent pathogen in HIV-positive admissions, both in unmatched and matched analyses. Crude mortality up to one year after ICU admission did not differ between HIV-positive and HIV-negative patients with pneumonia (either unmatched or matched) (Table 2).

**Table 2:** Baseline characteristics and outcome of unmatched and matched admissions for pneumonia stratified by HIV status

	Unmatched patients			Matched patients^a^	
	HIV-positive (n = 30)	HIV-negative (n = 1078)	*P*	HIV-negative (n = 90)	*P*
Demographics^b^					
Age, years, mean (SD)	51.5 (11.1)	60.6 (16.2)	0.001	50.8 (12.9)	0.83
Gender, male (%)	17 (77.3)	604 (64.9)	0.28	69 (84.1)	0.52
Race: white (%)	14 (63.6)	831 (89.3)	0.002	67 (81.7)	0.10
Comorbidities^b^					
Chronic renal insufficiency (%)	2 (9.1)	101 (10.8)	1	11 (13.4)	0.75
COPD (%)	4 (18.2)	178 (19.1)	1	11 (13.4)	0.73
Diabetes mellitus (%)	3 (13.6)	173 (18.6)	0.59	14 (17.1)	0.76
Hematologic malignancy (%)	5 (22.7)	73 (7.8)	0.03	10 (12.2)	0.30
Hypertension (%)	8 (36.4)	277 (29.8)	0.63	14 (17.1)	0.08
Liver cirrhosis (%)	1 (4.5)	16 (1.7)	0.32	2 (2.4)	1
Metastatic malignancy (%)	2 (9.1)	28 (3)	0.16	2 (2.4)	0.20
Non-metastatic malignancy (%)	2 (9.1)	96 (10.3)	1	5 (6.1)	0.64
Admissions					
Readmission (%)	8 (26.7)	147 (13.6)	0.06	8 (8.9)	0.03
Community-acquired pneumonia (%)	18 (60)	565 (52.4)	0.44	56 (62.2)	1
Severity of disease in first 24 hours					
SOFA score, median (IQR)	6 (4–8)	7 (3–10)	0.30	7 (4-9)	0.89
Organ failure (%)	884 (82)	25 (83.3)	1	81 (90)	0.70
Shock (%)	262 (24.3)	8 (26.7)	0.84	35 (38.9)	0.53
Supportive care in the first 24 hours					
Mechanical ventilation (%)	20 (66.7)	859 (79.7)	0.12	81 (90)	0.006
Renal replacement therapy (%)	2 (6.7)	69 (6.4)	1	8 (8.9)	1
Outcome					
Length of stay ICU, median days (IQR)	5 (1-15)	4 (2-9)	0.70	6 (3-11)	0.33
Organ failure during admission (%)	27 (90)	951 (88.2)	1	87 (96.7)	0.26
Shock during admission (%)	14 (46.7)	340 (31.5)	0.10	35 (38.9)	0.53
30-day mortality (%)^b^	4 (18.2)	244 (26.2)	0.47	19 (23.2)	0.79
60-day mortality (%)^b^	8 (36.4)	290 (31.1)	0.67	22 (26.8)	0.43
90-day mortality (%)^b^	9 (40.9)	319 (34.3)	0.66	22 (26.8)	0.30
1-year mortality (%)^b^	11 (50)	412 (44.3)	0.66	25 (30.5)	0.12

Results are presented as number (%) unless stated otherwise. ^a^Patients were matched for age, gender and race and compared with HIV-positive patients. ^b^Demographics and mortality data are given for the first ICU admission during the study period; readmissions were not included, resulting in analysis from 22 HIV-positive patients, 931 HIV-negative unmatched and 82 HIV-negative matched patients. *cART* combination antiretroviral therapy, *COPD* chronic obstructive pulmonary disease, *ICU* intensive care unit, *IQR* interquartile range, *SD* standard deviation, *SOFA* Sequential Organ Failure Assessment

**Table 3:** Causative pathogens in all unmatched and matched patients with pneumonia stratified according to HIV status

	Unmatched patients			Matched patients	
	HIV-positive (n = 30)	HIV-negative (n = 1078)	*P*	HIV-negative (n = 90)	*P*
Gram-positive bacteria (%)	8 (26.7)	216 (20)	0.52	18 (20.0)	0.44
*Streptococcus pneumoniae* (%)	4 (13.3)	71 (6.6)	0.27	5 (5.6)	0.22
*Streptococcus* species (%)	0	19 (1.8)	0.67	3 (3.3)	0.56
*Staphylococcus aureus* (%)	4 (13.3)	99 (9.2)	0.52	6 (6.7)	0.27
Other Gram-positive bacteria (%)	0	27 (2.5)	0.62	4 (4.4)	0.58
Gram-negative bacteria (%)	3 (10.0)	349 (32.4)	0.0075	36 (40.0)	0.011
*Haemophilus influenzae* (%)	0	70 (6.5)	0.26	9 (10)	0.11
*Escherichia coli* (%)	1 (3.3)	65 (6)	0.71	5 (5.6)	1
*Pseudomonas aeruginosa* (%)	0	64 (5.9)	0.25	6 (6.7)	0.33
*Klebsiella pneumoniae* (%)	1 (3.3)	29 (2.7)	1	3 (3.3)	1
*Enterobacter cloacae* (%)	0	25 (2.3)	0.63	1 (1.1)	1
*Stenotrophomonas maltophilia* (%)	1 (3.3)	11 (1)	0.28	1 (1.1)	0.44
Other Gram-negative bacteria (%)	0	85 (7.9)	0.18	11 (12.2)	0.06
Yeast/fungi (%)	6 (20.0)	84 (7.8)	0.036	18 (20.0)	1
*Aspergillus* species (%)	1 (3.3)	40 (3.7)	1	9 (10)	0.44
*Pneumocystis jirovecii* (%)	4 (13.3)	15 (1.4)	0.004	5 (5.6)	0.22
Other yeasts or fungi (%)	1 (3.3)	29 (2.7)	1	4 (4.4)	1
Viruses (%)	3 (10.0)	70 (6.5)	0.72	10 (11.1)	1
Influenza (%)	0	38 (3.5)	0.41	8 (8.9)	0.19
Cytomegalovirus (%)	2 (6.7)	5 (0.5)	0.019	0	0.049
Other viruses (%)	1 (3.3)	27 (2.5)	1	2 (2.2)	1
Atypical mycobacteria (%)	1 (3.3) ^a^	1 (0.1) ^b^	0.053	0	0.24
Unknown (%)	15 (50)	500 (46.4)	1	30 (33.3)	0.10

Results are presented as number (%). Percentages represent the proportion of pneumonia cases caused by the particular pathogen. Multiple causative pathogens were isolated in some patients with pneumonia. ^a^ *Mycobacterium avium*. ^b^ *Mycobacterium xenopi*

### Host response biomarkers in pneumosepsis

**Fig. 2:**
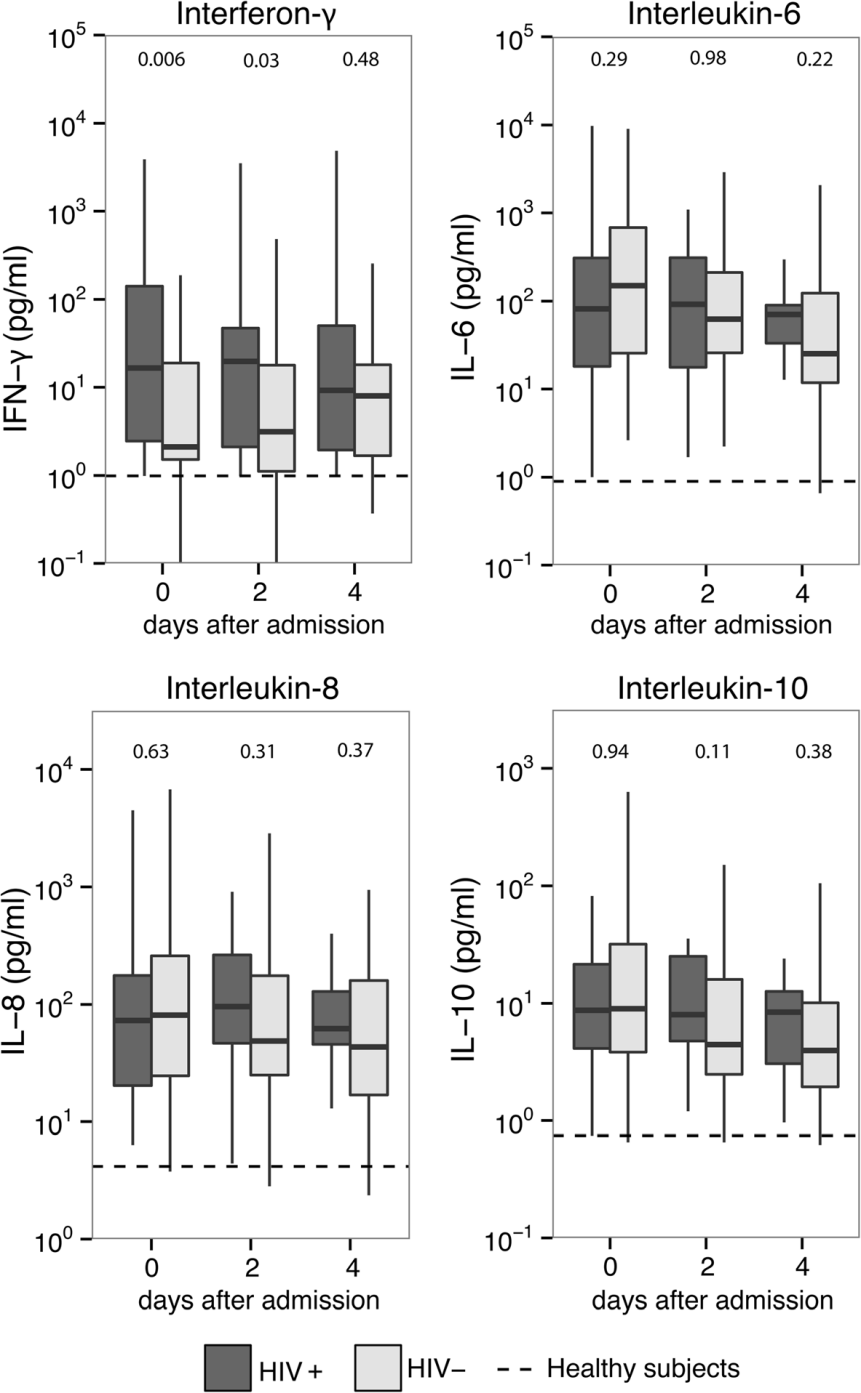
Cytokine levels in matched patients with pneumonia stratified according to HIV infection status. Plasma cytokine levels on days 0, 2 and 4 after intensive care unit admission. Data in box-and-whisker diagrams depict the median and lower quartile, upper quartile and respective 1.5 IQR as *whiskers* (as specified by Tukey). *Dashed lines* represent median in 27 healthy volunteers. *P* values are indicated for differences between HIV-positive and HIV negative-patients at each time point. *IFN* interferon, *IL* interleukin

**Fig. 3:**
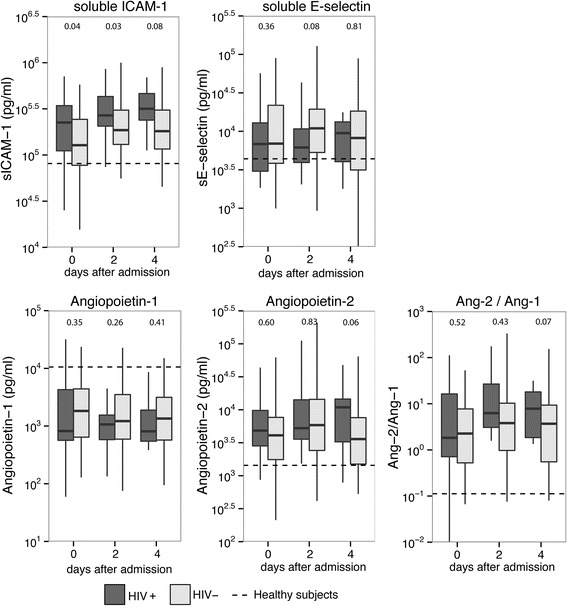
Endothelial cell activation markers in matched patients with pneumonia stratified according to HIV infection status. Plasma levels of soluble E-selectin, soluble intercellular adhesion molecule-1 (*ICAM*-*1*), angiopoietin-1 and angiopoietin-2 on days 0, 2 and 4 after intensive care unit admission. Data in box-and-whisker diagrams depict the median and lower quartile, upper quartile and the respective 1.5 IQR as *whisker*s (as specified by Tukey). *Dashed lines* represent median levels in 27 healthy volunteers. *P* values are indicated for differences between HIV-positive and HIV-negative patients at each time point

**Fig. 4:**
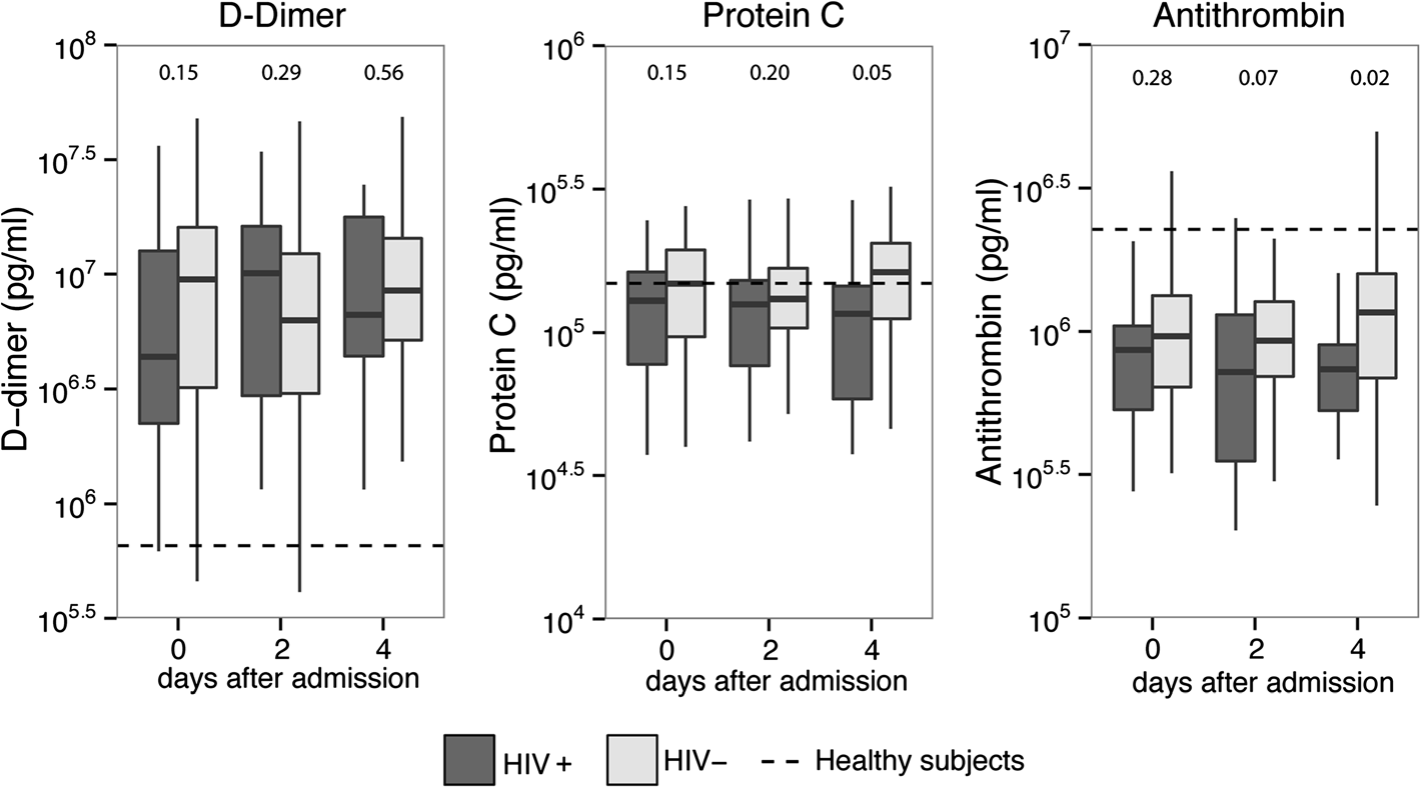
Coagulation activation markers in matched patients with pneumonia stratified according to HIV infection status. Plasma levels of D-dimer, protein C and antithrombin on days 0, 2 and 4 after intensive care unit admission. Data in box-and-whisker diagrams depict the median and lower quartile, upper quartile and the respective 1.5 IQR as *whiskers* (as specified by Tukey). *Dashed lines* represent median levels in 27 healthy volunteers. *P* values are indicated for differences between HIV-positive and HIV-negative patients at each time point

To obtain insight into the influence of HIV infection on the host response to sepsis we measured 14 biomarkers indicative of activation and/or deregulation of key pathways implicated in sepsis pathogenesis in 30 HIV-positive and 90 matched HIV-negative pneumosepsis admissions. As expected [20, 21], patients with sepsis displayed activation of the cytokine network (Fig. 2), the vascular endothelium (Fig. 3) and the coagulation system (Fig. 4). The concentrations of most host response biomarkers were similar in HIV-positive and HIV-negative admissions, except for IFN-γ and soluble ICAM-1, which were higher in HIV-positive admissions at days 0 and 2. These differences were no longer statistically significant when readmissions were excluded, thus analyzing only the first admission of unique patients (Additional file 2). The plasma concentrations of TNF-α, IL-1β and IL-13 were undetectable in the majority of patients and were not different between groups (data not shown).

## Discussion

We studied the impact of HIV infection on the presentation and outcome of sepsis, and particularly pneumosepsis. Our main findings were that disease severity and outcome are remarkably alike in HIV-positive and HIV-negative patients. In addition, plasma concentrations of biomarkers indicative of key host responses to sepsis were largely similar in HIV-positive and HIV-negative patients with pneumosepsis.

In previous studies, HIV/AIDS was independently associated with in-hospital mortality in ICU patients with sepsis [9–11]. These studies differ from the present investigation in patient selection [9, 10] and setting [11], which resulted in the inclusion of patients with more severe disease [9–11]. Standards of care for HIV patients have improved considerably over time and previous studies indicate that survival of critically ill HIV-infected patients continues to improve in the era of widespread availability of cART [22, 23]. Taken together these data suggest that access to care (e.g. cART and well-equipped ICUs) is an important factor in the outcome of sepsis in patients with HIV.

Pneumonia was a more frequent presentation in patients with sepsis and HIV co-infection. Previously, pneumonia was shown to be a major source of morbidity in HIV, even in patients with high CD4 cell counts [24]. Prior to the wide availability of cART, *P jirovecii* pneumonia was a common reason for ICU admission [25, 26]. In our cohort *P. jirovecii* was a more common pathogen in HIV-positive patients, in addition to CMV, but the numbers of these opportunistic pathogens were relatively small, with the majority of pneumonia cases being caused by bacterial pathogens, similar to HIV-negative patients. However, interpretation of these findings is limited by the fact that we were unable to identify a causative pathogen in approximately half of our patients and that the causative role of CMV ideally is confirmed by tissue examination, which was not routinely done. Although our data do not show differences in disease severity, mechanical ventilation in the first 24 hours after ICU admission was applied less frequently in HIV-positive patients with pneumonia.

We sought to examine the effect of HIV infection on the host response in a matched subgroup of patients with pneumosepsis. Previous reports on the host response to sepsis in adult patients with HIV are limited to two investigations from Brazil in which plasma cytokine levels were studied [27, 28]. Few differences according to HIV status were observed, but one of these studies reported higher plasma IL-10 in the presence of unaltered IL-6 among HIV-positive patients with sepsis [28]. Notably, these encompassed only patients with advanced AIDS-defining disease, HIV-negative control groups unmatched for age and site of infection, and very high mortality rates (around 50 %) [27, 28].

We analyzed host response biomarkers in a relatively homogeneous cohort of patients with pneumosepsis matched for age, gender and white race, and found no differences in the plasma levels of proinflammatory and anti-inflammatory cytokines, with the sole exception of IFN-γ. The main producers of IFN-γ are activated natural killer (NK) cells, T-helper-1 cells, and cytotoxic T cells [29]. Our finding of higher plasma IFN-ɣ in patients with sepsis and HIV co-infection, which was sustained up to two days after ICU admission, is remarkable, as patients with HIV generally have reduced numbers of circulating NK cells and T-helper-1 cells [13]. Furthermore, NK cells from untreated HIV patients released less IFN-γ in response to bacterial stimulation than NK cells from HIV-negative controls [30]. The clinical and biological relevance of (modestly) elevated IFN-γ levels in HIV-infected patients with sepsis remains to be established.

Chronic HIV infection is associated with endothelial cell activation and damage [13], responses that are almost invariably also found in patients with sepsis [31]. In Malawian children with severe bacterial infection, a greater increase in plasma angiopoietin-2, an angiogenic peptide that increases endothelial activation and vascular permeability, was observed in patients with HIV co-infection [32]. In our adult ICU patients with pneumosepsis, plasma levels of specific endothelial cell activation markers (angiopoietin-1 and -2, and soluble E-selectin) did not differ according to HIV status. We did observe higher levels of soluble ICAM-1 in HIV-positive patients with pneumosepsis, which can be shed by both endothelial cells and leukocytes. HIV infection can stimulate the release of exosomes containing ADAM metallopeptidase domain 17 (ADAM17), the cleaving protease for ICAM-1, which promotes ICAM-1 shedding [33]. Increased levels of IFN-ɣ, as observed in our study, may also contribute to the release of soluble ICAM-1 [34]. Although previous studies have described a procoagulant state in patients with HIV [35, 36], plasma levels of D-dimer, protein C and antithrombin were similar in HIV-positive and HIV-negative patients with pneumosepsis. These results indicate that HIV infection has no additive effect on activation of the vascular endothelium and coagulation in critically ill patients with pneumonia.

Our study has strengths and limitations. We prospectively analyzed all consecutive patients admitted with sepsis to two ICUs during a 2.5-year period. Nonetheless, the number of HIV-positive patients with sepsis was limited, precluding stratification according to HIV disease progression. HIV testing is not standard for all ICU patients; thus, our control group may have contained cases with unrecognized HIV infection. However, The Netherlands has a low HIV prevalence of around 0.2 %, so this is unlikely to influence our results [37]. This study was conducted in two academic ICUs and therefore generalization of results should be done with caution. Sepsis was defined using the 2001 consensus definition [18]; the vast majority of included patients had a SOFA score ≥2 at ICU admission, which approximates the recently updated consensus definitions for sepsis [38]. Finally, known HIV infection may lead to selection bias in admittance to ICU and/or the extent of aggressive therapy.

## Conclusions

Pneumonia is the main cause of sepsis in HIV-positive ICU patients, and is more frequent compared to patients without HIV infection. Otherwise, our results indicate that in a high-resource setting with excellent access to care and HIV treatment, HIV infection has little, if any, influence on the clinical and pathophysiological course of sepsis requiring ICU admission. These findings support the notion that the presence of HIV co-infection should not play a major role in the decision whether or not to admit critically ill patients with sepsis to the ICU.

## Additional files

Additional file 1: Characteristics of HIV-positive patients with pneumosepsis with and without readmission. (DOCX 17 kb)
Additional file 2: Plasma biomarkers in the matched pneumosepsis cohort during the first admission (readmissions excluded). (DOCX 17 kb)

## Abbreviations

cART: combination antiretroviral therapy
CMV: cytomegalovirus
HIV: human immunodeficiency virus
ICAM: intercellular adhesion molecule
ICU: Intensive Care Unit
IFN: interferon
IL: interleukin
SOFA: Sequential Organ Failure Assessment
TNF: tumor necrosis factor

## References

[1] Kaplan JE, Hanson D, Dworkin MS, Frederick T, Bertolli J, Lindegren ML (2000). Epidemiology of human immunodeficiency virus-associated opportunistic infections in the United States in the era of highly active antiretroviral therapy. Clin Infect Dis.

[2] Huson MA, Stolp SM, van der Poll T, Grobusch MP (2014). Community-acquired bacterial bloodstream infections in HIV-infected patients: a systematic review. Clin Infect Dis.

[3] Japiassu AM, Amancio RT, Mesquita EC, Medeiros DM, Bernal HB, Nunes EP (2010). Sepsis is a major determinant of outcome in critically ill HIV/AIDS patients. Crit Care.

[4] Casalino E, Wolff M, Ravaud P, Choquet C, Bruneel F, Regnier B (2004). Impact of HAART advent on admission patterns and survival in HIV-infected patients admitted to an intensive care unit. AIDS.

[5] Huang L, Quartin A, Jones D, Havlir DV (2006). Intensive care of patients with HIV infection. N Engl J Med.

[6] Kim JH, Psevdos G, Gonzalez E, Singh S, Kilayko MC, Sharp V (2013). All-cause mortality in hospitalized HIV-infected patients at an acute tertiary care hospital with a comprehensive outpatient HIV care program in New York City in the era of highly active antiretroviral therapy (HAART). Infection.

[7] Medrano J, Alvaro-Meca A, Boyer A, Jimenez-Sousa MA, Resino S (2014). Mortality of patients infected with HIV in the intensive care unit (2005 through 2010): significant role of chronic hepatitis C and severe sepsis. Crit Care.

[8] Barbier F, Roux A, Canet E, Martel-Samb P, Aegerter P, Wolff M (2014). Temporal trends in critical events complicating HIV infection: 1999-2010 multicentre cohort study in France. Intensive Care Med.

[9] Mrus JM, Braun L, Yi MS, Linde-Zwirble WT, Johnston JA (2005). Impact of HIV/AIDS on care and outcomes of severe sepsis. Crit Care.

[10] Tolsma V, Schwebel C, Azoulay E, Darmon M, Souweine B, Vesin A (2014). Sepsis severe or septic shock: outcome according to immune status and immunodeficiency profile. Chest.

[11] Cribbs SK, Tse C, Andrews J, Shenvi N, Martin GS (2015). Characteristics and outcomes of HIV-infected patients with severe sepsis: continued risk in the post-highly active antiretroviral therapy era. Crit Care Med.

[12] Angus DC, van der Poll T (2013). Severe sepsis and septic shock. N Engl J Med.

[13] Huson MA, Grobusch MP, van der Poll T (2015). The effect of HIV infection on the host response to bacterial sepsis. Lancet Infect Dis.

[14] Klein Klouwenberg PM, Ong DS, Bos LD, de Beer FM, van Hooijdonk RT, Huson MA (2013). Interobserver agreement of Centers for Disease Control and Prevention criteria for classifying infections in critically ill patients. Crit Care Med.

[15] Kaukonen KM, Bailey M, Suzuki S, Pilcher D, Bellomo R (2014). Mortality related to severe sepsis and septic shock among critically ill patients in Australia and New Zealand, 2000-2012. JAMA.

[16] Garner JS, Jarvis WR, Emori TG, Horan TC, Hughes JM (1988). CDC definitions for nosocomial infections, 1988. Am J Infect Control.

[17] Calandra T, Cohen J, International Sepsis Forum Definition of Infection in the ICUCC (2005). The international sepsis forum consensus conference on definitions of infection in the intensive care unit. Crit Care Med.

[18] Levy MM, Fink MP, Marshall JC, Abraham E, Angus D, Cook D (2003). 2001 SCCM/ESICM/ACCP/ATS/SIS International Sepsis Definitions Conference. Intensive Care Med.

[19] R core team. A language and environment for statistical computing. http://www.R-project.org/. Accessed 5 Jul 2015.

[20] Pierrakos C, Vincent JL (2010). Sepsis biomarkers: a review. Crit Care.

[21] Parlato M, Cavaillon JM (2015). Host response biomarkers in the diagnosis of sepsis: a general overview. Methods Mol Biol.

[22] Powell K, Davis JL, Morris AM, Chi A, Bensley MR, Huang L (2009). Survival for patients With HIV admitted to the ICU continues to improve in the current era of combination antiretroviral therapy. Chest.

[23] Huson MA, Bakhshi-Raiez F, Grobusch MP, de Jonge E, de Keizer NF, van der Poll T (2016). Characteristics and outcome of AIDS patients in Dutch intensive care units between 1997 and 2014. Crit Care Med.

[24] Gordin FM, Roediger MP, Girard PM, Lundgren JD, Miro JM, Palfreeman A (2008). Pneumonia in HIV-infected persons: increased risk with cigarette smoking and treatment interruption. Am J Respir Crit Care Med.

[25] Akgun KM, Pisani M, Crothers K (2011). The changing epidemiology of HIV-infected patients in the intensive care unit. J Intensive Care Med.

[26] Corona A, Raimondi F (2009). Caring for HIV-infected patients in the ICU in the highly active antiretroviral therapy era. Curr HIV Res.

[27] Amancio RT, Japiassu AM, Gomes RN, Mesquita EC, Assis EF, Medeiros DM (2013). The innate immune response in HIV/AIDS septic shock patients: a comparative study. PLoS One.

[28] Silva JM, dos Santos Sde S (2013). Sepsis in AIDS patients: clinical, etiological and inflammatory characteristics. J Int AIDS Soc.

[29] Schoenborn JR, Wilson CB (2007). Regulation of interferon-gamma during innate and adaptive immune responses. Adv Immunol.

[30] Dillon SM, Lee EJ, Bramante JM, Barker E, Wilson CC (2014). The natural killer cell interferon-gamma response to bacteria is diminished in untreated HIV-1 infection and defects persist despite viral suppression. J Acquir Immune Defic Syndr.

[31] Opal SM, van der Poll T (2015). Endothelial barrier dysfunction in septic shock. J Intern Med.

[32] Mankhambo LA, Banda DL, Group IPDS, Jeffers G, White SA, Balmer P (2010). The role of angiogenic factors in predicting clinical outcome in severe bacterial infection in Malawian children. Crit Care.

[33] Lee JH, Wittki S, Brau T, Dreyer FS, Kratzel K, Dindorf J (2013). HIV Nef, paxillin, and Pak1/2 regulate activation and secretion of TACE/ADAM10 proteases. Mol Cell.

[34] Witkowska AM, Borawska MH (2004). Soluble intercellular adhesion molecule-1 (sICAM-1): an overview. Eur Cytokine Netw.

[35] Haugaard AK, Lund TT, Birch C, Rönsholt F, Trøseid M, Ullum H, Gerstoft J, Johansson PI, Nielsen SD, Ostrowski SR (2013). Discrepant coagulation profile in HIV infection: elevated D-dimer but impaired platelet aggregation and clot initiation. AIDS.

[36] Jong E, Louw S, van Gorp EC, Meijers JC, ten Cate H, Jacobson BF (2010). The effect of initiating combined antiretroviral therapy on endothelial cell activation and coagulation markers in South African HIV-infected individuals. Thromb Haemost.

[37] Stichting HIV monitoring. Monitoring report 2014; Monitoring of human immunodeficiency virus (HIV) infection in the Netherlands 2014. http://www.hiv-monitoring.nl. Accessed 5 Jan 2016.

[38] Singer M, Deutschman CS, Seymour CW, Shankar-Hari M, Annane D, Bauer M (2016). The third international consensus definitions for sepsis and septic shock (Sepsis-3). JAMA.

