# Supplementary material for: Impact of HIV infection on the presentation, outcome and host response in patients admitted to the intensive care unit with sepsis; a case control study
Source: Crit Care. 2016 Oct 10;20:322. doi: 10.1186/s13054-016-1469-0 (PMC5056483; doi:10.1186/s13054-016-1469-0)
Supplement: Additional file 1: — Characteristics of HIV-positive patients with pneumosepsis with and without readmission. (DOCX 17 kb) [file 13054_2016_1469_MOESM1_ESM.docx]

**Additional file 1: Characteristics of HIV positive pneumosepsis patients with and without readmission**

|  | First admission of patients without readmission  n=17 | First admission of patients with a readmission during follow up  n=5 | Readmission  n=8 |
| --- | --- | --- | --- |
| **Demographics** |  |  |  |
| Age, years, mean [SD] | 51.6 [11.6] | 51 [10.4] | 54.4 [10.2] |
| Gender, male (%) | 12 (70.6) | 5 (100) | 8 (100) |
| Race: white (%) | 9 (52.9) | 5 (100) | 8 (100) |
| **Characteristics of infection** |  |  |  |
| Community-acquired (%) | 12 (70.6) | 3 (60) | 3 (37.5) |
| **Comorbidities** |  |  |  |
| Chronic renal insufficiency (%) | 2 (11.8) | -- | 2 (25) |
| COPD (%) | 2 (11.8) | 2 (40) | 2 (25) |
| Diabetes mellitus (%) | 1 (5.9) | 2 (40) | 2 (25) |
| Hematologic malignancy (%) | 4 (23.5) | 1 (20) | 1 (12.5) |
| Hypertension (%) | 6 (35.3) | 2 (40) | 2 (25) |
| Liver cirrhosis (%) | 1 (5.9) | -- | -- |
| Metastatic malignancy (%) | 1 (5.9) | 1 (20) | 2 (25) |
| Non-metastatic malignancy (%) | 1 (5.9) | 1 (20) | 1 (12.5) |
| **Severity of disease in first 24 hours** |  |  |  |
| SOFA score, median [IQR] | 7 [3-8] | 10 [7-12] | 7 [4-11] |
| Organ failure (%) | 14 (82.4) | 5 (100) | 6 (75) |
| Shock (%) | 5 (29.4) | 1 (20) | 2 (25) |
| **HIV disease severity and treatment** |  |  |  |
| CD4 count, cells/mm^3^), median [IQR]^a^ | 50 [32-363] | 214 [19-921] | 80 [28-220] |
| Viral load, cp/ml, median [IQR]^b^ | 20132[40-0.71*10^6^] | 50 [43-1.95*10^6^] | 114 [50-503] |
| On cART (%) | 10 (59) | 4 (80) | 7 (88) |

^a^ CD4 counts were available for 28 admissions

^b^ Viral loads were available for 29 admissions
