# Supplementary material for: Impact of HIV infection on the presentation, outcome and host response in patients admitted to the intensive care unit with sepsis; a case control study
Source: Crit Care. 2016 Oct 10;20:322. doi: 10.1186/s13054-016-1469-0 (PMC5056483; doi:10.1186/s13054-016-1469-0)
Supplement: Additional file 2: — Plasma biomarkers in the matched pneumosepsis cohort during the first admission (readmissions excluded). (DOCX 17 kb) [file 13054_2016_1469_MOESM2_ESM.docx]

**Additional file 2:**  **Plasma biomarkers in the matched pneumosepsis cohort during the first admission (readmissions excluded).**

|  |  | Admission (day 0) | | |  | Day 2 | | |  | Day 4 | | |
| --- | --- | --- | --- | --- | --- | --- | --- | --- | --- | --- | --- | --- |
|  |  | HIV +  N = 19 ^a^ | HIV -  N = 71 | *p* |  | HIV +  N = 21 | HIV -  N = 78 | *p* |  | HIV +  N = 9 | HIV -  N = 50 | *p* |
| **Pro- and anti-inflammatory cytokines** | | | | | | | | | | | | |
| Interferon-γ (pg/ml) |  | 9.2 [2-55.4] | 2.7 [1.7-18.9] | 0.08 |  | 12.7 [2-49.3] | 5.1 [1.7-28.8] | 0.13 |  | 9.2 [2.1-50.2] | 8 [1.8-17.2] | 0.54 |
| Interleukin-6 (pg/ml) |  | 107 [14.2-423.3] | 181.6 [27.8-730.6] | 0.24 |  | 79.6 [16.9-414] | 92.8 [34.6-233.1] | 0.79 |  | 69.7 [33.2-74.6] | 25.2 [12.8-119.6] | 0.58 |
| Interleukin-8 (pg/ml) |  | 95.2 [20.8-176.5] | 85.6 [25.9-280.2] | 0.59 |  | 91.8 [38.3-247.6] | 49.4 [25.3-195.6] | 0.65 |  | 70.1 [36.8-128.9] | 43.4 [16.6-153] | 0.52 |
| Interleukin-10 (pg/ml) |  | 9.1 [4.5-23.7] | 11.2 [4.2-37.8] | 0.93 |  | 9.4 [4.6-25.2] | 5.3 [2.9-21.1] | 0.22 |  | 8.4 [5.4-12.6] | 4.3 [2-12.8] | 0.35 |
| **Endothelial cell activation** | | | | | | | | | | | | |
| Soluble ICAM-1 (ng/ml) ^b^ |  | 223.2 [86.1-341.5] | 127.8 [77.3-243.6] | 0.27 |  | 235.3 [188.5-489.3] | 183.3 [128.5-306.4] | 0.15 |  | 339.7 [112.3-531.8] | 176 [109.2-300.6] | 0.24 |
| Soluble E-Selectin (ng/ml) |  | 4.7 [3-17.1] | 8 [4.5-22.6] | 0.31 |  | 8.3 [4.1-17.1] | 11.5 [6.5-19.4] | 0.25 |  | 10.2 [6-13.3] | 9.1 [3.3-18.6] | 0.89 |
| Angiopoietin-1 (ng/ml) |  | 0.8 [0.6-4.4] | 2.3 [0.7-4.8] | 0.25 |  | 1 [0.6-1.7] | 1.6 [0.7-4.3] | 0.20 |  | 0.8 [0.7-2.3] | 1.4 [0.7-3.2] | 0.45 |
| Angiopoietin-2 (ng/ml) |  | 4.3 [2-5.5] | 4.1 [1.6-7.7] | 0.77 |  | 5 [3.8-11.4] | 6.1 [2.5-15.4] | 0.89 |  | 4.8 [3.3-11.5] | 3.6 [1.6-7.9] | 0.31 |
| Ang-2/Ang-1 ratio |  | 1.8 [0.5-12.9] | 1.7 [0.5-7.4] | 0.71 |  | 5.6 [2.7-14.5] | 3.7 [0.7-10.6] | 0.61 |  | 4.7 [1.6-8.9] | 3.7 [0.5-6.1] | 0.36 |
| **Coagulation activation** | | | | | | | | | | | | |
| D-dimer (µg/ml) |  | 4.4 [1.6-12.5] | 9.5 [3.9-16] | 0.11 |  | 11.9 [3.6-16.4] | 6.3 [3-12.2] | 0.14 |  | 7.2 [4.4-17.8] | 8.7 [5.1-15.1] | 0.78 |
| Protein C (ng/ml) |  | 129.1 [68.6-156.4] | 144.2 [96.7-190.5] | 0.07 |  | 105.2 [72.8-144] | 126.9 [103-165.4] | 0.11 |  | 89.3 [58.6-144.9] | 152.9 [110.1-200.1] | 0.05 |
| Antithrombin (ng/ml) |  | 862.1 [579.6-1108.1] | 918.7 [618.1-1292.3] | 0.62 |  | 750.8 [354.9-1157.8] | 902.3 [692.7-1251.8] | 0.20 |  | 744.4 [528.6-898.4] | 1026.6 [658.8-1527.3] | 0.07 |

Plasma levels on day 0, 2 and 4 after intensive care unit admission. Results are presented as medians and interquartile ranges.

Abbreviation: ICAM, intercellular adhesion molecule.

^a^ Number of patients of whom plasma was available for measurement of biomarkers.

^b^ Soluble intercellular adhesion molecule-1 also originates from leukocytes.
